# An evaluation of whether propensity score adjustment can remove the self-selection bias inherent to web panel surveys addressing sensitive health behaviours

**DOI:** 10.1186/s12874-020-01134-4

**Published:** 2020-10-08

**Authors:** Andrew Copas, Sarah Burkill, Fred Conrad, Mick P. Couper, Bob Erens

**Affiliations:** 1 Institute for Global Health, University College London, London, UK; 2 Saw Swee Hock School of Public Health, National University of Singapore, Singapore, Singapore; 3 Survey Research Center, University of Michigan, Ann Arbor, Michigan, USA; 4 Department of Health Services Research and Policy, London School of Hygiene and Tropical Medicine, London, UK

**Keywords:** Internet survey, Web survey, Survey methods, Sampling bias, Selection bias, Sexual behaviour, Propensity score adjustment

## Abstract

**Background:**

In health research, population estimates are generally obtained from probability-based surveys. In market research surveys are frequently conducted from volunteer web panels. Propensity score adjustment (PSA) is often used at analysis to try to remove bias in the web survey, but empirical evidence of its effectiveness is mixed. We assess the ability of PSA to remove bias in the context of sensitive sexual health research and the potential of web panel surveys to replace or supplement probability surveys.

**Methods:**

Four web panel surveys asked a subset of questions from the third British National Survey of Sexual Attitudes and Lifestyles (Natsal-3). Five propensity scores were generated for each web survey. The scores were developed from progressively larger sets of variables, beginning with demographic variables only and ending with demographic, sexual identity, lifestyle, attitudinal and sexual behaviour variables together. The surveys were weighted to match Natsal-3 based on propensity score quintiles. The performance of each survey and weighting was assessed by calculating the average ‘absolute’ odds ratio (inverse of the odds ratio if less than 1) across 22 pre-specified sexual behaviour outcomes of interest comparing the weighted web survey with Natsal-3. The average standard error across odds ratios was examined to assess the impact of weighting upon variance.

**Results:**

Propensity weighting reduced bias relative to Natsal-3 as more variables were added for males, but had little effect for females, and variance increased for some surveys. Surveys with more biased estimates before propensity weighting showed greater reduction in bias from adjustment. Inconsistencies in performance were evident across surveys and outcomes. For most surveys and outcomes any reduction in bias was only partial and for some outcomes the bias increased.

**Conclusions:**

Even after propensity weighting using a rich range of information, including some sexual behaviour variables, some bias remained and variance increased for some web surveys. Whilst our findings support the use of PSA for web panel surveys, the reduction in bias is likely to be partial and unpredictable, consistent with the findings from market research. Our results do not support the use of volunteer web panels to generate unbiased population health estimates.

## Background

Health surveys conducted by government and academic researchers have traditionally used probability sampling of addresses and data collection facilitated by an interviewer who visits the address, for example the third British National Survey of Sexual Attitudes and Lifestyles (Natsal-3) [1]. Such surveys are considered broadly representative of the general population and a ‘gold standard’ method, although they have some limitations such as poor coverage of hard-to-reach groups and susceptibility to nonresponse bias. By contrast in market research surveys are now widely conducted by inviting members of volunteer web panels to participate. Web panels provide comparatively cheap and quick data collection on individuals whose basic demographic characteristics are already known [2] and hence are appealing in principle to health researchers as a method by which to replace or supplement a traditional survey if concerns over bias can be resolved.

Population internet coverage is now high and increasing in many countries, for example it is estimated to be over 90% among 16–44 year olds in the UK [3]. Therefore almost all the general population is eligible to join a web panel, though some coverage bias may still be evident since the declining group without internet access is becoming increasingly different to those with access [4–6]. Furthermore a web panel survey is typically representative of the general population by design in terms of certain demographic characteristics which are used to set quotas for the sample. Nevertheless the primary concern about web panel surveys is that they are non-probability and likely to suffer from self-selection bias, arising because individuals who join web panels differ from those who do not. Nonresponse bias can also be substantial since the response rate within a panel for a particular survey is typically very low [7].

We commissioned four different web panel surveys in which participants were asked a range of sexual behaviour and other questions identical to those asked in Natsal-3. Two web surveys followed a standard methodology and two other web surveys set additional quotas using data from Natsal-3 in the hope this would reduce any bias. In earlier work, by comparing estimates for key behaviours and attitudes with Natsal-3, we established however that the bias in the web panel surveys was too substantial for their use to be recommended in this topic area, even when additional quotas were set [8]. In this paper we investigate the ability of propensity score adjustment (PSA) to reduce bias at the analysis stage.

Propensity scores have been proposed for a range of uses in the analysis of observational epidemiological studies [9, 10]. In market research PSA is widely used for web panel surveys [11], although attempts at empirical validation of the reduction in bias have had mixed findings [12–14] and a recent task force reported that PSA should not be expected to remove all bias [15]. The basis of PSA is to identify a set of auxiliary variables, available in both the web survey and the census (or a large probability sample reference survey), that reflect key dimensions in which web survey participants differ from the general population. In this context the propensity score measures the propensity to be in the web survey rather than in the census, or reference survey, as applicable. Auxiliary variables may be selected because they are also associated with the outcomes of interest. PSA then ensures the distribution of auxiliary variables in the web panel survey broadly matches that in the census or reference survey, for example through weighting [16, 17]. The assumption of PSA is that this matching removes the self-selection bias in the web survey [18, 19]. In market research key auxiliary variables reflecting attitudes or behaviours known as “webographics” are used [20, 21]. For estimation of wages Steinmetz et al. used data concerning self-reported quality of life and quality of working life [12], whereas for estimation of a range of outcomes Mercer et al. used measures of political attitudes and engagement [13]. In market research, reference surveys have been conducted to allow PSA [11, 22]. Web panel surveys together with PSA can however be used more widely and cheaply if a reference survey specific to each research topic is not required, i.e. if auxiliary variables measured in the census or multipurpose reference surveys are sufficient. PSA may decrease the precision of estimates, so this must be counter-balanced against any reduction in bias [23].

This study makes an important contribution to the ongoing debate as to whether PSA is effective in reducing or eliminating the bias that is inherent in web panel surveys. We assess the issues in health where web surveys are currently little used, but in principle there could be considerable demand, and makes a unique contribution by addressing a research topic of high sensitivity. Multiple web surveys are used which allows us to investigate consistency of PSA performance and Natsal-3 allows us to investigate whether a topic-specific reference survey is required for the adjustment.

## Methods

### Surveys

Four web surveys (each with at least 2000 participants) were conducted between May and July 2012 by three different well known and reputable market research companies based in the UK, see earlier work for details [8]. Two web surveys used basic quotas (identified as B-1 and B-2 in this paper) defined by age, sex, region and partnership status. Two surveys used modified quotas (M-1 and M-2), which were set using additional variables not normally used such as age finished full-time education and frequency of drinking alcohol. However the market research companies involved struggled to fill the modified quotas. Neither survey could find enough people who finished their education before age 17 years, and the survey set additional quota failed to meet all of them falling short of large (≥4 person) households, infrequent drinkers of alcohol (less than once a week), and those expressing intolerance of same-sex relationships. These quotas had to be relaxed in order to ensure we gained enough responses within the given time frame.

Natsal-3 was undertaken between September 2010 and August 2012 and 15,162 participants aged 16–74, resident in Britain participated. Eight thousand nine hundred sixty-nine of these participants were in our age group of interest (18–44 years), see [1] for further details.

### Propensity score model selection

For each web survey separately we built multiple propensity score models in successive stages, including in the model selection process at the first stage only demographic variables, and then progressively more variables at each subsequent stage including sexual identity (stage 2), non-sexual behaviours (stage 3), attitudes (stage 4) and finally the richest model at the stage 5 in which key selected sexual behaviour variables could be selected. For each stage a backward stepwise model selection process was conducted based on logistic regression using survey participation (web or Natsal-3) as the outcome variable, and the selection process was conducted separately for males and females. At the second and later stages those variables selected at the previous stage were included in the model with certainty and further variables were selected only if they were statistically significant (*p* < 0.05) after adjusting for the variables already included. Overall there were 5 stages, and 5 corresponding PSAs for each survey and gender. The regression models were fitted to weighted data. The standard weights for Natsal-3 were used, based on selection weighting and then post-stratification by age, sex and region to the 2011 census distribution [1]. For participants in each web survey, ‘initial’ weights were based on post-stratification by age and sex to the 2011 census. Table 1 show the variables included in the process at each stage of model building, and the variables selected for the propensity score model for each of the 4 web surveys by gender.

**Table 1:**
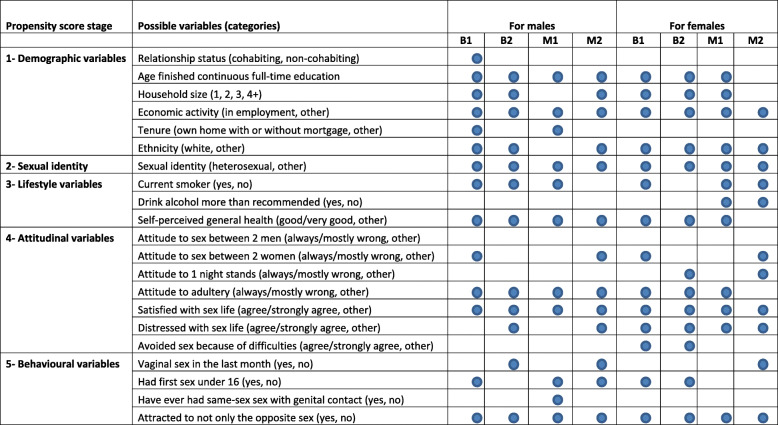
Variables selected by PSA stage: for males and females

### Propensity score weights calculation

For each web survey, stage, and gender, all participants from both web survey and Natsal-3 were given a propensity score which was the fitted probability from the corresponding logistic regression model. For weighting, five equally sized subclasses were defined by the quintiles of the score of participants for both surveys combined, as commonly recommended [4, 21] and in the original development of propensity score methods by Rosenbaum and Rubin [9, 10]. For each subclass the ‘PSA ratio’ was calculated equal to the ratio of the proportion of Natsal-3 in the subclass to the proportion of web survey participants in the subclass, each proportion calculated using the same weights used to fit the propensity regression models (standard weights for Natsal-3 and ‘initial’ weights for each web survey). The PSA weight for each web survey participant at each stage was the product of their initial weight and the PSA ratio for the stage corresponding to the participant’s subclass. This ensured that for each web survey at each stage, by each gender, the distribution across the subclasses weighted by the PSA weights matched Natsal-3 under standard weighting. If a participant had a missing propensity score due to missing data for any of the variables in the model, their age-within sex weight was used.

### Analysis and variance estimation

Twenty-two outcome variables were included in the analysis (see Tables 2 and 3). These key outcomes used in initial Natsal-3 publications were defined *a priori* by the set used in our previous paper looking into the impact of quota setting on web panel estimates [8], after excluding those lifestyle variables, attitudes and behaviours which were available for selection into the propensity score models at stages 3–5. These outcomes were binary; categorical outcome variables were recoded to binary in order to avoid multiple outcomes for the same variable. We obtained an odds ratio (OR) for each outcome, for the web survey relative to Natsal-3, under PSA weighting at each stage for each web survey in comparison to Natsal-3 (under standard weighting) from binary logistic regression. As a measure of the performance of the web survey (and PSA weighting) relative to Natsal-3, average absolute ORs were then calculated across the 22 outcomes, where by absolute OR we mean the OR, or 1/OR if the OR is less than 1 [8]. Average absolute ORs were calculated at each of the 5 stages in order to ascertain whether estimates were improved when PSA was applied. As we view Natsal-3 as minimally biased the absolute ORs are viewed as a measure of bias in the web surveys relative to the Natsal-3 estimates.

When PSA is used through weighting, the variance of the estimates typically increases with the number of variables included in the propensity score model since this makes the weights more variable, so there is a trade-off between bias and precision [4, 21]. The weights used for PSA have uncertainty because they are created using estimates from a model, and to account for this and sampling variability we used replicate weights to obtain a bootstrapped standard error (SE). The focus of the paper is the comparison of each web survey with Natsal-3 and how PSA weighting affects these comparisons. For PSA Natsal-3 is viewed as a reference standard and hence the uncertainty in Natsal-3 estimates is ignored. For each web survey and each PSA weighting stage we calculate the SE for the absolute OR corresponding to each outcome. We use the average of this SE across all 22 outcomes as our measure of precision and to see graphically how this varies across the five stages of PSA weighting for each web survey. We note that larger ORs have larger SEs in general and hence also interpret any changes to the SE from PSA in light also of how the absolute ORs are changed.

All analyses were conducted using STATA 12.

## Results

**Fig. 1:**
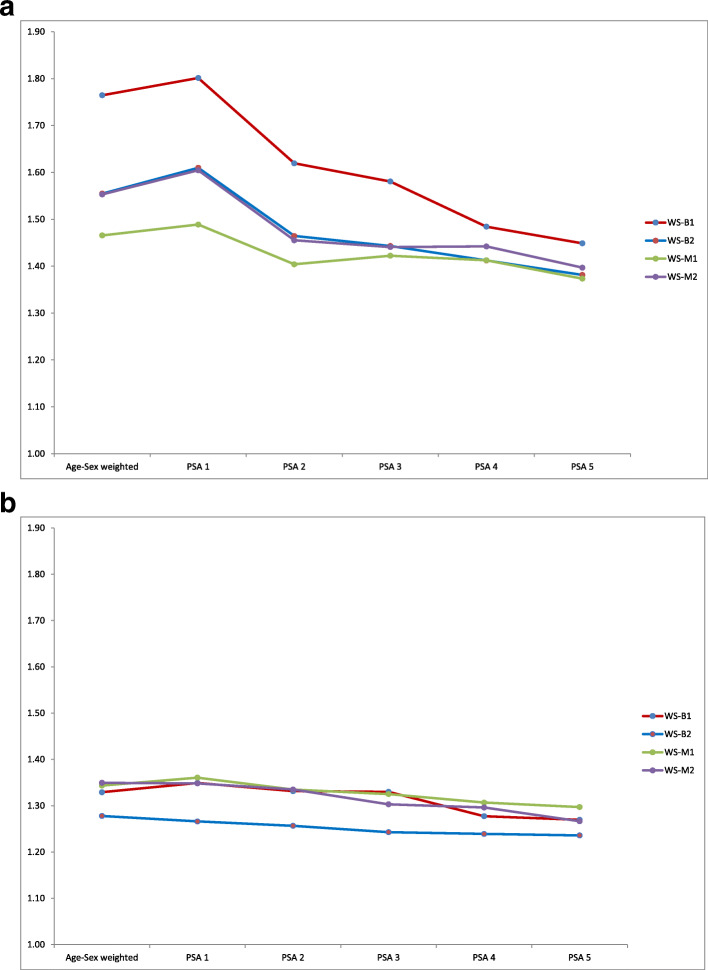
**a** Average absolute odds ratio across 22 outcomes: males. **b**. Average absolute odds ratio across 22 outcomes: females

To assess the performance of PSA, we show average absolute ORs relative to Natsal-3 for each of the 4 web surveys across the 22 selected outcomes separately for males (Fig. 1a) and females (Fig. 1b). It seems that under PSA weighting the average absolute OR decreases with increasing stage, i.e. as more variables are used in the PSA. This reduction in bias is however modest for female participants. The impact of PSA seems to differ according to web survey, with those web surveys starting with the highest average absolute ORs when the age-sex weight is applied showing the largest decrease in bias under PSA. This results in a partial ‘convergence’ in the absolute bias between the 4 web surveys with each successive PSA stage, at least for males. It can also be seen that there is more bias for males even after stage 5 PSA than for females when just the age-sex weight is used. The smaller improvements seen under PSA for females than males may be partly attributable to less bias before PSA.

We intentionally included sexual identity as the only additional variable at stage 2 so as to assess the impact of this variable of special interest because it is a demographic factor and yet closely related to sexual behaviour. For all web surveys and for both males and females this variable was selected to contribute to the PSA weighting (see Table 1). For males, compared to other stages, bias was most clearly reduced moving from stage 1 to stage 2 (Fig. 1a). However improvements across subsequent stages were also substantial for some web surveys. For females, there does not seem to be any single PSA stage which substantially improved results across all web surveys.

To assess whether PSA weighting has achieved broadly unbiased estimates, and to illustrate the variation in PSA performance across outcomes, we also present estimates for each outcome in the ‘best performing’ survey for males and females in Tables 2 and 3 respectively. For women we select B-2 as best performing survey because it has the smallest average absolute OR at all PSA stages (Fig. 1b), and for men we also select B-2 because it has joint smallest average absolute OR at stage 5 PSA and the best reduction in bias from PSA (Fig. 1a). To aid interpretation we have informally described bias relative to Natsal-3 as small where the absolute OR is less than 1.25, moderate where it is in the range 1.25–1.67, and large if greater than 1.67.

**Table 2:** Estimated prevalence of each of 22 key preselected behaviours in males in the best performing survey (WS-B2), before and after PSA weighting in five stages and in Natsal-3 for comparison, odds ratios and bias assessment

	Proportions by weighting scheme and in Natsal-3							Odds ratios relative to Natsal-3		Bias^3^ relative to Natsal-3	
Behaviour	Age-sex	PSA 1	PSA 2	PSA 3	PSA 4	PSA 5	Natsal-3	Age-sex	PSA5	Age-sex	PSA5
‘Binge’ drinks once a week or more^1^	0.170	0.168	0.163	0.167	0.162	0.165	0.214	0.75	0.73	moderate	moderate
Both equally willing at first sex	0.882	0.886	0.888	0.890	0.901	0.899	0.911	0.73	0.87	moderate	small
Contraception used at first sex	0.864	0.864	0.872	0.868	0.872	0.864	0.820	1.39	1.39	moderate	moderate
First sex at about the right time	0.608	0.623	0.622	0.633	0.648	0.641	0.743	0.54	0.62	large	moderate
Experience not all with opposite sex	0.162	0.155	0.119	0.119	0.110	0.089	0.072	2.49	1.26	large	moderate
Had heterosexual oral sex in last year	0.715	0.727	0.742	0.747	0.746	0.747	0.793	0.65	0.77	moderate	moderate
Had heterosexual anal sex in last year	0.204	0.203	0.202	0.211	0.197	0.202	0.176	1.20	1.19	small	small
Sex without condom in last 4 weeks	0.769	0.779	0.781	0.776	0.791	0.790	0.749	1.12	1.26	small	moderate
1+ same gender partners last 5 years	0.079	0.076	0.046	0.050	0.045	0.036	0.030	2.77	1.21	large	small
Attended STI clinic in last 5 years	0.143	0.141	0.128	0.128	0.125	0.134	0.187	0.73	0.67	moderate	moderate
Attended STI clinic in last year	0.058	0.055	0.046	0.046	0.046	0.049	0.069	0.83	0.70	small	moderate
Ever STI diagnosis	0.123	0.126	0.119	0.116	0.105	0.113	0.133	0.91	0.83	small	small
Ever taken illicit drugs	0.446	0.459	0.456	0.451	0.448	0.446	0.523	0.73	0.73	moderate	moderate
Ever taken cannabis	0.407	0.419	0.416	0.410	0.410	0.409	0.491	0.71	0.72	moderate	moderate
1+ new heterosexual partners last year	0.247	0.236	0.232	0.238	0.222	0.222	0.274	0.87	0.76	small	moderate
0 heterosexual partners lifetime	0.116	0.099	0.088	0.087	0.086	0.089	0.060	2.06	1.53	large	moderate
0 heterosexual partners last 5 years	0.143	0.124	0.107	0.107	0.106	0.109	0.077	2.00	1.47	large	moderate
0 heterosexual partners last year	0.200	0.183	0.165	0.167	0.157	0.161	0.112	1.98	1.52	large	moderate
Heterosexual sex 5+ occasions last 4 weeks	0.511	0.520	0.519	0.528	0.546	0.550	0.464	1.21	1.41	small	moderate
Ever paid for heterosexual sex	0.132	0.129	0.128	0.125	0.117	0.117	0.104	1.31	1.14	moderate	small
Had a sexual function problem in last year^2^	0.437	0.433	0.423	0.411	0.397	0.396	0.395	1.19	1.00	small	small
Easy to talk to parent(s) about sex at age 14	0.161	0.153	0.151	0.160	0.162	0.167	0.184	0.85	0.89	small	small

1. Defined as drinking 6+ units of alcohol; 2. Based on half the sample only; 3. Small bias is an odds ratio relative to Natsal-3 in the range 0.8–1.25, moderate bias is in the range 0.6–0.8 or 1.25–1.67, large bias otherwise

**Table 3:** Estimated prevalence of each of 22 key preselected behaviours in females in the best performing survey (WS-B2), before and after PSA weighting in five stages and in Natsal-3 for comparison, odds ratios and bias assessment

	Proportions by weighting scheme and in Natsal-3							Odds ratios relative to Natsal-3		Bias^3^ relative to Natsal-3	
Behaviour	Age-sex	PSA 1	PSA 2	PSA 3	PSA 4	PSA 5	Natsal-3	Age-sex	PSA5	Age-sex	PSA5
‘Binge’ drinks once a week or more^1^	0.098	0.099	0.095	0.098	0.090	0.092	0.118	0.81	0.76	small	moderate
Both equally willing at first sex	0.791	0.789	0.788	0.794	0.800	0.805	0.821	0.83	0.90	small	small
Contraception used at first sex	0.876	0.870	0.868	0.868	0.871	0.875	0.856	1.19	1.18	small	small
First sex at about the right time	0.566	0.554	0.554	0.563	0.567	0.577	0.644	0.72	0.75	moderate	moderate
Experience not all with opposite sex	0.245	0.233	0.223	0.218	0.207	0.192	0.159	1.72	1.26	large	moderate
Had heterosexual oral sex in last year	0.705	0.714	0.717	0.720	0.739	0.745	0.770	0.71	0.87	moderate	small
Had heterosexual anal sex in last year	0.166	0.168	0.168	0.164	0.172	0.169	0.154	1.09	1.12	small	small
Sex without condom in last 4 weeks	0.789	0.797	0.799	0.798	0.794	0.793	0.800	0.93	0.96	small	small
1+ same gender partners last 5 years	0.064	0.062	0.055	0.052	0.052	0.047	0.049	1.33	0.96	moderate	small
Attended STI clinic in last 5 years	0.184	0.179	0.178	0.179	0.177	0.174	0.203	0.89	0.83	small	small
Attended STI clinic in last year	0.060	0.057	0.056	0.056	0.058	0.056	0.085	0.69	0.64	moderate	moderate
Ever STI diagnosis	0.172	0.176	0.175	0.173	0.163	0.158	0.188	0.90	0.81	small	small
Ever taken illicit drugs	0.406	0.392	0.388	0.380	0.373	0.360	0.385	1.09	0.90	small	small
Ever taken cannabis	0.368	0.353	0.351	0.344	0.339	0.327	0.355	1.06	0.88	small	small
1+ new heterosexual partners last year	0.174	0.168	0.169	0.170	0.166	0.167	0.218	0.76	0.72	moderate	moderate
0 heterosexual partners lifetime	0.059	0.058	0.055	0.057	0.053	0.054	0.045	1.33	1.21	moderate	small
0 heterosexual partners last 5 years	0.099	0.094	0.090	0.090	0.084	0.082	0.064	1.61	1.31	moderate	moderate
0 heterosexual partners last year	0.177	0.168	0.163	0.161	0.148	0.144	0.107	1.79	1.40	large	moderate
Heterosexual sex 5+ occasions last 4 weeks	0.527	0.526	0.524	0.523	0.540	0.542	0.452	1.35	1.43	moderate	moderate
Ever paid for heterosexual sex	0.147	0.155	0.157	0.152	0.150	0.146	0.148	0.99	0.98	small	small
Had a sexual function problem in last year^2^	0.506	0.496	0.492	0.485	0.460	0.449	0.483	1.10	0.87	small	small
Easy to talk to parent(s) about sex at age 14	0.149	0.148	0.145	0.146	0.154	0.154	0.252	0.52	0.54	large	large

1. Defined as drinking 6+ units of alcohol; 2. Based on half the sample only; 3. Small bias is an odds ratio relative to Natsal-3 in the range 0.8–1.25, moderate bias is in the range 0.6–0.8 or 1.25–1.67, large bias otherwise

We see that a substantial proportion of estimates across the 22 outcomes remained noticeably biased under PSA weighting compared to Natsal-3, particularly for males, even by stage 5 and having selected the best performing surveys. For males (Table 2) stage 5 PSA weighting did reduce bias for all 6 outcomes affected by large bias under only age-sex weighting, to a moderate level for 5 outcomes and a small level for one (1+ same gender partners in the last 5 years). For outcomes affected by small or moderate bias under age-sex weighting the performance of PSA weighting was less impressive. Most outcomes affected by moderate bias retained moderate bias under PSA weighting and for 4 outcomes affected by small bias this increased to moderate under PSA weighting (e.g. heterosexual sex on 5+ occasions in the last 4 weeks). Of 22 outcomes 15 were moderately biased after stage 5 PSA weighting. For females (Table 3) bias was also reduced to a degree for all 3 outcomes affected by large bias under age-sex weighting, though this remained large for one outcome (easy to talk to parents about sex at age 14). Bias was reduced to small for 3 of the 8 outcomes affected by moderate bias under age-sex weighting. For only one outcome did bias increase to moderate from small through PSA weighting. Even in this survey however bias was judged to be moderate or large for 9 of 22 outcomes after stage 5 weighting.

**Fig. 2:**
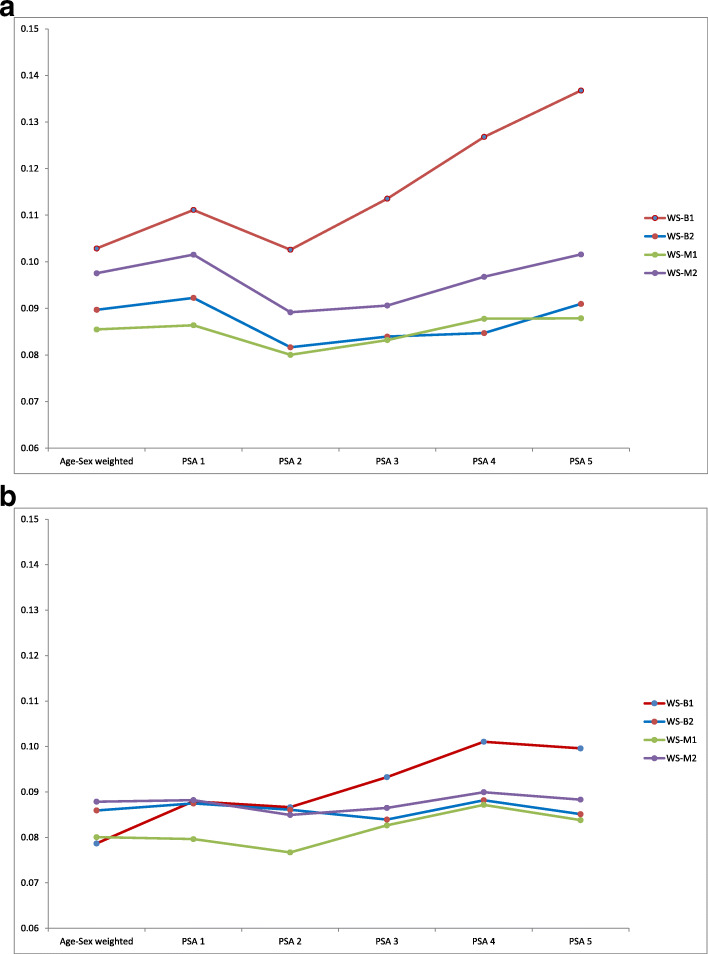
**a**. Average standard error of odds ratio across 22 outcomes: males. **b**. Average standard error of odds ratio across 22 outcomes: females

For both males and females there is some evidence of the expected trade-off between bias and precision. The average SE is seen to increase across the stages of PSA (Fig. 2a and b) for two of the four surveys in women, and among men for survey B-1 which has the greatest reduction in bias. In other surveys the SE is little changed but since the absolute OR is reduced (towards 1) by PSA an unchanged SE can be seen also as a decrease in precision.

## Discussion

This investigation aimed to assess whether PSA is an effective means of removing the self-selection and other biases of web panel surveys whilst maintaining an acceptable degree of precision. The results suggest that PSA can reduce bias, i.e. estimates became closer to the reference survey Natsal-3. However these improvements were not consistent across variables or web surveys, and in some cases estimates moved further away from the reference. The reductions in bias were not sufficiently large and consistent in general for estimates to be seen as broadly unbiased, even after the final stage PSA which was based in part upon data for outcomes of interest obtained from a topic-specific reference survey. A decrease in precision from PSA was suggested in all surveys and very clear for two surveys among women and one for men.

Our findings suggest that PSA may improve estimates more when these estimates, before PSA, are more heavily biased. This may arise because a larger number of variables are selected by the PSA modelling process, meaning a larger number of variables contribute to the propensity score weighting. For those surveys which performed best under only age-sex weighting, we saw only minimal improvements from PSA.

For men we saw a ‘convergence’ in bias between web surveys, as measured by the average absolute odds ratio, over successive PSA stages as more variables are included in the adjustment. This however does not imply any convergence in the bias for individual outcomes, and web surveys still differed in an unpredictable way. The findings suggest that PSA at analysis can achieve some of the bias reduction hoped for by setting modified quotas at the design stage, and this is to be expected. However it is important to remember that PSA reduces bias by weighting to match the reference survey or census which is less efficient (less precision) than matching by design.

We found that using only demographic variables in the PSA, as available from the census or a general reference survey, did not begin to counteract the bias in the web surveys (in fact for most surveys we saw bias increase). This has also been found in market research and led to the use of “webographics” including key attitude variables [21]. In our study, because we had a topic specific reference survey, we were able to also use attitudes relating to the topics of interest and even some behaviours as auxiliary variables but this did not fully remove bias. Nevertheless reductions in bias from PSA were evident in all web surveys to a greater or lesser degree, and this increased as more auxiliary variables were used in the PSA. Sexual identity was a helpful auxiliary variable and may be viewed as a webographic in the context of sexual health. Homosexual and bisexual identities were much more common in web panel survey participants and also associated with outcomes of interest. If we added more and more outcomes of interest as auxiliary variables in the PSA, estimates might continue to improve and eventually be deemed sufficiently unbiased for practical use, but then the web surveys would contribute little additional information as for all these key outcomes they would simply duplicate the information from the reference survey. We cannot exclude the possibility that researchers might over time be able to develop “health webographics” that could be used with volunteer web panel surveys addressing health research topics and included in one or more reference surveys. However our findings give no encouragement to believe such webographics would be fully effective in reducing bias through PSA, and their effectiveness even in market research where they are most developed is unclear [12]. An empirical investigation similar to ours but in the context of politics in the USA had similar findings to ours [13], whilst others conclude adjustment for any observable characteristics will be generally insufficient [6].

An important limitation is that, though we view Natsal-3 as superior to the web surveys because of its probability sampling and treat it as a broadly unbiased ‘benchmark’, it is not impossible that the web surveys might provide more accurate estimates for some behaviours. The greater anonymity afforded to web panel participants could result in the higher levels of disclosure seen for some sensitive behaviours in some surveys [24]. Indeed findings from analysis of a web follow up study to Natsal-3, in which the same respondents were asked the same questions roughly a month after their initial interview, suggest possible mode effects for some behaviours [25]. Furthermore PSA is designed to account for differences between the web panel survey participants and the general population and is not expected to remove any reporting bias arising for example from mode effects. We have not investigated how PSA affects bias in the estimation of associations, and in further work we shall investigate whether PSA is more effective for that purpose than for estimating population prevalence.

Although probability sampling and face-to-face interviewing are still the preference of government and academic researchers, decreasing response rates and increasing costs make the prospect of using alternative methods appealing [26]. Web panel surveys offer in principle significant cost savings if used in a standalone fashion. There are also other possibilities to use web panels in conjunction with probability surveys. Firstly web panels might provide ‘boosts’ of additional participants for either the whole population or for subgroups of particular interest that may be hard to find or uncommon in the general population. Secondly, if the probability survey is general or multipurpose in its nature then web panels may be used to provide detailed information for topics of special interest. An alternative approach is to either directly combine probability sampling with survey self-completion online, possibly allowing greater anonymity as might suit surveys of sensitive topics, or to invite participants in a previous probability survey to complete an online survey.

## Conclusions

In earlier work [8] we established that setting quotas for web panel surveys alone does not provide unbiased estimates in our context of the sexual behaviour of the British general population. Therefore here we investigated whether PSA based on either standard demographic factors or topic-specific information can consistently reduce most or all of the bias affecting web panel surveys. At least in our context, where moderate changes in behaviour over time would be of great interest, we have considered the bias seen even after extensive PSA to be unacceptable. Different degrees of bias may be tolerated in other contexts. Nevertheless our main conclusion which supports evidence from other contexts [e.g. 13] is that volunteer web panels, even if conducted in conjunction with a reference survey so that the richest possible PSA is used, cannot be recommended in general to provide accurate population estimates.

## Data Availability

The Natsal-3 data have been archived at the UK Data Archive: http://www.data-archive.ac.uk/

## Abbreviations

Natsal-3: Third British National Survey of Sexual Attitudes and Lifestyles
OR: Odds ratio
PS: Propensity score
PSA: Propensity score adjustment
SE: Standard error
